# The Role of Ubiquitination and Sumoylation in Diabetic Nephropathy

**DOI:** 10.1155/2014/160692

**Published:** 2014-06-04

**Authors:** Chenlin Gao, Wei Huang, Keizo Kanasaki, Yong Xu

**Affiliations:** ^1^Department of Endocrinology, Affiliated Hospital of Luzhou Medical College, Luzhou, Sichuan 646000, China; ^2^Division of Diabetology and Endocrinology, Kanazawa Medical University, Uchinada, Ishikawa 920-0293, Japan

## Abstract

Diabetic nephropathy (DN) is a common and characteristic microvascular complication of diabetes; the mechanisms that cause DN have not been clarified, and the epigenetic mechanism was promised in the pathology of DN. Furthermore, ubiquitination and small ubiquitin-like modifier (SUMO) were involved in the progression of DN. MG132, as a ubiquitin proteasome, could improve renal injury by regulating several signaling pathways, such as NF-**κ**B, TGF-**β**, Nrf2-oxidative stress, and MAPK. In this review, we summarize how ubiquitination and sumoylation may contribute to the pathology of DN, which may be a potential treatment strategy of DN.

## 1. Introduction

Diabetic nephropathy (DN) is a common and characteristic microvascular complication of diabetes. About 30–40% of patients with type 1 diabetes (T1DM) and 20–30% of patients with type 2 diabetes (T2DM) would develop DN after the disease duration of 15–30 years [1, 2]. The morbidity of DN is rising year by year with the increasing incidence and prevalence of diabetes dramatically and disproportionately, and the pathogenesis of DN has not been completely clarified; its treatment is limited, unsatisfactory, and expensive [3]. DN patients always have a poor prognosis which is a leading cause of end-stage renal disease and a contributor to significant morbidity and mortality in patients with diabetes [4].

Hyperglycemia, oxidative stress, advanced glycation end products, and angiotensin II can lead to the occurrence of DN by activating the transforming growth factor-*β* (TGF-*β*) signaling pathway, nuclear factor *κ*B (NF-*κ*B) signaling pathway, Nrf2-oxidative stress, and mitogen-activated protein kinase (MAPK) pathway. Recent studies found the ubiquitin proteasome system and small ubiquitin-like modifier (SUMO) can be involved in these pathways through the regulation of protein ubiquitination and SUMO [5]. However, the relationship between ubiquitination, SUMO, and DN remains to be elucidated, although several recent papers have suggested that ubiquitination and SUMO are involved in the pathogenesis of DN. In this review, we summarize and discuss recent findings on the role of ubiquitination and SUMO in diabetic nephropathy.

## 2. Ubiquitination

**Figure 1:**
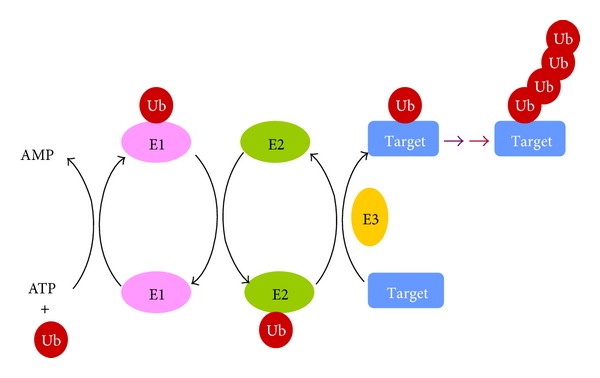
Protein ubiquitination: ubiquitination is a multistep sequential process, which begins with the activation of ubiquitin by the ATP-dependent activating enzyme E1 followed by transfer of ubiquitin to the ubiquitin-carrier protein E2, forming a thioester linked E2-ubiquitin (E2-Ub) intermediate. The substrate-recruiting ubiquitin-protein ligase E3 interacts with the E2-Ub allowing for the transfer of ubiquitin to the target.

Ubiquitin is a small, highly conserved, ubiquitously expressed protein with immensely important and diverse regulatory functions. Ubiquitination is a multistep process involving the sequential action of three enzymes: ATP-dependent activating enzyme E1, ubiquitin-carrier protein E2, and ubiquitin-protein ligase E3 enzyme. The conjugation process begins with the activation of ubiquitin by the E1 followed by transfer of ubiquitin to the E2, forming a thioester linked E2-ubiquitin (E2-Ub) intermediate. The substrate-recruiting E3 interacts with the E2-Ub allowing for the transfer of ubiquitin to the target (Figure 1) [6]. There is usually a single E1 and there are many E2 proteins and multiple specific E3 proteins. Each E3 protein appears to be responsible for the specific ubiquitin-protein ligation [7–9]. A well-studied function of ubiquitin is its role in selective proteolysis by the ubiquitin-proteasome system (UPS), which serves as a mechanism to modify cellular functions and protein function such as cell signaling, protein trafficking, DNA repair, chromatin modifications, cell-cycle progression, and cell death. The highly specific modification of USP to numerous proteins is made possible by the abundance and diversity of ubiquitin ligases, especially the large number of E3.

The grinder UPS machinery contains a barrel-shaped 20S proteolytic core particle (CP) with 28 subunits which was complemented with either the 19S regulatory complex and/or the 11S regulatory heptamers [10]. The proteolytic sites of the core particle are blocked within the proteasome barrel, which ensures inertness of the proteolytic activity. So, without the regulatory subunits, the 20S complex is known as an inherently repressed enzyme. Attainability and delivery of target proteins within the 20S barrel for degradation are dependent on the function of proteasome regulators. Selecting target proteins to ubiquitination and degradation is fulfilled by specific ubiquitin ligase (E2 and E3 enzymes) [11]. This selection is balanced by interactions among different target substrates, ubiquitin ligases, ubiquitin ligase adapter proteins, and chaperons. And the timely and selective ubiquitination and degradation of redundant, dysfunctional and/or abnormal proteins are crucial for normal cellular physiology. Any delay, disturbance in the process of degradation by the UPS will break the homeostasis between cellular and tissue [12]. It is involved in a great deal of cell signal pathways which participate in the progression of DN.

The inhibition of systemic proteasome could ameliorate renal pathologies, so the ability to modulate UPS activity has a good effect on battling nephropathies. Studies found that members of UPS, cullin-1, cullin-3, and the 11S proteasome regulators PA28-*β* and PA28-g are definitely upregulated in intraglomerular capillaries of mice with DN [13].

## 3. SUMO

A more recently identified transient protein modification is the attachment of a SUMO peptide, the process of which is often referred to as SUMOylation. SUMO polypeptides are approximately 18% identical to ubiquitin at the amino acid sequence level and their three-dimensional (3D) structural folds are highly similar to those of ubiquitin. First reported by two research groups in late 1996 and early 1997, sumoylation is now known to modify various eukaryotic proteins in organisms ranging from yeast to humans. Sumoylation has also been shown to occur in signaling pathways. Initially found only within the nucleus, SUMOylated proteins have now been discovered in the cytoplasm, including the mitochondria and the plasma membrane [14]. SUMO, both monomeric and polymeric addition to substrates, has been documented, but distinct biological functions have only recently been ascribed to these modifications [15].

Mammalian cells express three major SUMO hypotypes, called SUMO-1, SUMO-2, and SUMO-3. SUMO-1 shares 50% sequence identity with SUMO-2 and SUMO-3, while SUMO-2/3 shares a 95% identity. Also, SUMO-2/3 possess consensus SUMOylation sites at their N-terminal tails that allow formation of poly-SUMO chains, in contrast to SUMO-1, where these sites are absent, along with the capacity for chain formation. SUMO-4 is the most recently identified gene and has an 86% similarity to SUMO-2. Its mRNA transcripts are mainly present in kidney, lymph system, and spleen but show limited expression compared to the other SUMlO species [16]. Since no native SUMO-4 protein has yet been detected in any tissue, it has been suggested that SUMO-4 might be an expressed pseudogene [17].

SUMO conjugation necessitates an enzymatic cascade resembling that of ubiquitination. The mature form of SUMO is activated in an ATP-dependent manner by an E1-activating enzyme, which consists of an SAE1-SAE2 heterodimer. Activated SUMO is transferred to Ubc9 (E2 conjugase) to form a thioester bond and is subsequently attached to the e-amino group of a lysine residue in the target substrates [18]. The mature form of SUMO is activated in an ATP-dependent manner by an E1-activating enzyme, which consists of an SAE1-SAE2 heterodimer. Activated SUMO is transferred to Ubc9 (E2 conjugase) to form a thioester bond and is subsequently attached to the e-amino group of a lysine residue in the target substrates. Even though Ubc9 itself associates with SUMO and transfers SUMO to targets, specific SUMO E3 ligases are required for efficient modification. Several classes of SUMO ligases have been identified: the family of protein inhibitor of activated STAT (PIAS; PIAS1, PIAS2(x), PIAS3, PIAS4(y)), Polycomb-2 protein (Pc2), and RanBP2/Nup358, a component of the nuclear pore complex [19].

SUMO attachment is a reversible and highly transient modification. The same enzymes that facilitate the initial maturation of SUMO molecules also catalyze the cleavage from their substrates. To conjugate SUMO to the substrates, the proform of SUMO needs to be cleaved by sentrin/SUMO-specific proteases (SENPs) that hydrolyse C-terminal end to expose Gly-Gly motif [20]. SENP not only has a hydrolase activity but also has an isopeptidase activity, which regulates deconjugation of sumoylated substrates. Six human SENP family proteins, SENPs 1–3 and 5–7, have been shown to be SUMO-specific proteases. Unlike the enzymes catalyzing SUMO attachment, SUMO proteases show little similarity to the equivalent enzymes in the ubiquitin pathway but appear closely related to viral proteases. The differential subcellular localisation of the SENP proteins, most likely dictated by nonconserved N-terminal sequences, is thought to provide the specificity for the SUMO-substrate complexes they regulate.

## 4. Ubiquitination and SUMO

An association between SUMOylation and the ubiquitin pathway is not surprising because of the close relation between the two proteins. Since SUMO peptides can use the same lysine residues as ubiquitin, the SUMOylation of substrate protein may lead to protection from degradation in ubiquitin pathway. This interaction may have a competitive basis, in which SUMO and ubiquitin contend for attachment at the same lysine residue on the substrate and which will result in the conferral of opposite or different fates. For example, inhibitor of nuclear factor-*κ*B (I*κ*B*α*) is degraded upon its phosphorylation-induced ubiquitination on Lys21 and Lys22, whereas SUMOylation on Lys21 stabilizes the protein [21]. Similarly, SUMOylation on Lys277 and Lys309 translocates NEMO (I*κ*B kinase regulatory particle) to the nucleus, whereas phosphorylation-induced ubiquitination of the same residues translocates NEMO back to the cytoplasm [22].

By contrast to the competitive nature of the SUMO/ubiquitin interplay described above for other proteins, these posttranslational modifications can also cooperate to produce a similar outcome. In a sequential manner, SUMO modification can serve as a targeting signal for the ubiquitin proteasome pathway. The concept of a mechanism for proteolytic regulation of proteins modified by poly-SUMO signals has emerged from the finding that SUMO-2/3 polychain conjugates accumulate when mammalian cells are exposed to proteasome inhibitors. This finding led to the identification of a new class of SUMO-targeted ubiquitin ligases in yeast and, subsequently, the human orthologue. As exemplified in humans, SUMO-Ub chains synthesized by RNF4 target PML (promyelocytic leukemia protein) for proteasomal degradation [23]. However, it remained unclear whether SUMO-Ub chains are recognized as distinct signals by hybrid chain-specific receptors or by receptors recognizing ubiquitin alone.

The connections between the SUMO and ubiquitin pathways become even more intricate when considering the fact that the enzymes of one pathway can be regulated by the other. For example, the stability of two SUMO-specific proteases, SENP2 and 3, is regulated by the ubiquitin proteasome system. Moreover, the ubiquitin-conjugating enzyme, E2-25k, is inactivated by its SUMOylation. Mdm2 is an ubiquitin ligase E3, it can be self-ubiquitinated, leading to its degradation, which can be prevented by SUMOylation at the same lysine residue [24].

## 5. Ubiquitination, SUMO in the Progression of DN

Diabetic nephropathy (DN) is a common and serious microvascular complication of diabetes mellitus, which is the leading cause of end-stage renal disease and renal failure in western countries. Recent studies have shown that inflammation is a key link in the development of early DN [25]. Disturbance of glucose metabolism and abnormal hemodynamics can trigger inflammation and infiltration of mononuclear macrophage and excessive secretion of inflammatory factors could be detected in kidney tissue in early stage, which would cause kidney damage and accelerate renal fibrosis. Studies found ubiquitination and SUMO are involved in the incidence of DN widely [26]. Altering the activity of UPS and SUMO may contribute to the development of microvascular complications of diabetes. Moreover, studies found the ubiquitin fusion protein UbA52 increased significantly in urine of diabetes mellitus with macro- or microalbuminuria (DM-NP) patients by proteomic analysis. The alteration of UbA52 concentrations would be a marker for diagnosing and predicting the clinical course of DN [27].

### 5.1. Ubiquitination, SUMO, and Nuclear Factor Kappa B (NF-*κ*B)

**Figure 2:**
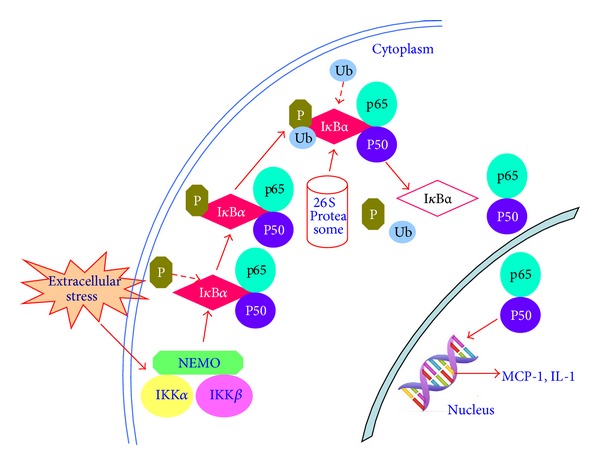
The process of activation of NF-*κ*B. In the resting state, NF-*κ*B combined with suppressed protein I*κ*B to compose a heterotrimer in the cytoplasm by an inactive form that would stop NF-*κ*B from entering the nucleus. When cell was stimulated by extracellular stress, the subunit of I kappa B kinase (I*κ*B kinase, IKK), IKK*β* would be activated first, and then the I*κ*B would be phosphorylated, and the lysine residues in this domain cause ubiquitination. After the ubiquitination of I*κ*B, it would be degraded by the 26S proteasome of UPP. At last, the heterotrimer dissociate, so that the heterodimers of p50-p65 exhibit the activity of NF-*κ*B and enter in the nucleus to be involved in gene transcription and protein synthesis.

Nuclear factor kappa B (NF-*κ*B) pathway is the main pathway in inflammation of DN, and it comprises a family of transcription factors and plays a central regulatory role in expression of various inflammatory cytokines involved in the occurrence of DN [28]. The extensive researches discovered NF-*κ*B family of transcription factors were regulated by the UPS system [26]. In the resting state, NF-*κ*B combined with suppressed protein I*κ*B to compose a heterotrimer in the cytoplasm by an inactive form that would stop NF-*κ*B from entering the nucleus. The mechanism of activation of NF-*κ*B is a complex process usually caused by the infection of bacteria, virus, and inflammation factor including TNF-a, IL-1*β*, and LPS (lipopolysaccharide) [29, 30]. First, the subunit of I kappa B kinase (I*κ*B kinase, IKK), IKK*β*, is phosphorylated. Then in I*κ*B N terminal regulatory domain of the heterotrimer of NF-*κ*B, Ser32/36 can also be phosphorylated, and the lysine residues in this domain occur ubiquitination. After the ubiquitination of I*κ*B, it would be degraded by the 26S proteasome of UPP. At last, the heterotrimer dissociate, so that the heterodimers of p50-p65 exhibit the activity of NF-*κ*B and enter in the nucleus to be involved in gene transcription and protein synthesis (Figure 2) [5].

Ubiquitination of I*κ*B and NF-*κ*B dissociation is a key step in NF-*κ*B activation, which would be involved in the occurrence and development of inflammation. Increased expression of ubiquitin was involved in the activation of NF-*κ*B in the atherosclerotic plaque of T2DM, which would influence the stability of atherosclerotic plaque [31]. Compared to the normal rats the expression of NF-*κ*B increased significantly in nucleus with DM, and the ubiquitin expression was increased sharply in cytoplasm of glomerular cells. So ubiquitin mediated the inflammatory pathway of NF-*κ*B which would lead to renal injury in diabetic rats [32].

The present study found that multiple signal transduction molecules of NF-*κ*B pathway, such as I*κ*B*α*, NEMO, RelA, and P100, can be modified by SUMO [21]. Because the relationship between SUMO and ubiquitination is not clear, so the role of sumoylation in the regulation of NF-*κ*B signal has been controversial. Examples include SUMO-1 modification of I*κ*B*α*, the main inhibitor of canonical NF-*κ*B dimers, which prevents signal-induced ubiquitination and degradation of I*κ*B*α* and thus limits NF-*κ*B activation. I*κ*B*α* conjugated to the SUMO-1, which is resistant to signal-induced degradation. SUMO-1 modified I*κ*B*α* remains associated with NF-*κ*B and thus overexpression of SUMO-1 inhibits the signal-induced activation of NF-*κ*B-dependent transcription. Reconstitution of the conjugation reaction with highly purified proteins demonstrated that in the presence of a novel E1 SUMO-1 activating enzyme, Ubc9 directly conjugated SUMO-1 to I*κ*B*α* on residues K21 and K22, which are also used for ubiquitin modification. Thus, while ubiquitination targets proteins for rapid degradation, SUMO-1 modification acts antagonistically to generate proteins resistant to degradation [33].

The activation of NF-*κ*B by cytokines under hyperglycemic conditions is a potential mechanism for complications in diabetes. The renal expression of TNF-*α*, NF-*κ*B (p65), I*κ*B*α*, and SUMO4 was significantly higher in diabetic GK rats. Translocation of NF-*κ*B (p65) and I*κ*B*α* into the nucleus was observed, and the expression of SUMO4 and I*κ*B*α* was upregulated in the glomerular endothelial cells. SUMO4 was localized in the cytoplasm and nucleus, while I*κ*B*α* was predominantly located in the nucleus after stimulation with TNF-*α* [34]. These showed that cytokines have a unique effect in regulating the sumoylation of NF-*κ*B and SUMO4 plays a role in regulating NF-*κ*B signaling in glomerular cells [34].

It is not clear whether SUMO-2/3 follows the same functional role as SUMO-1 and SUMO-4 during the activation of NF-*κ*B. Studies results showed the expression of SUMO1 and SUMO2/3 under high glucose was obviously enhanced. High glucose induced degradation of I*κ*B*α* and activation of NF-*κ*B. SUMO2/3-induced modification of I*κ*B*α* was only affected by high glucose [35]. Whether SUMO E3 is involved in the sumoylation of I*κ*-B*α* and whether a specific SENP removes the SUMO moiety from I*κ*-B*α* have yet to be determined. Other researches showed that the ectopic expression of mouse SUMO-2 inhibited IL-12 secretion by blocking the translocation of the p65 subunit of NF-*κ*B into the nucleus, which led to the polarization of naive CD4+ T cells to T helper 2 (Th2) shift in vitro, and indicate that high glucose may activate NF-*κ*B inflammatory signaling through I*κ*B*α* sumoylation and the functional role of SUMO-2/3 in the regulation of NF-*κ*B activity was conserved during evolution [36].

Similarly, posttranslational modification of p100 by SUMO is a determining factor for stimuli-induced p100 processing, which is the primary step in activating the alternative NF-*κ*B, and blocking SUMOylation of p100 would inhibit ultimate activation of the alternative NF-*κ*B pathway [37]. The SUMO-1 modification of NEMO (NF-*κ*B essential modulator) would mediate NF-*κ*B activation in response to genotoxic stress. Now, specific SUMO ligase or SUMO-specific protease (SENP) in SUMOylation or deSUMOylation for respective substrate remains unclear; the critical ligase is defined as PIASy [38].

A recent study reported the identification of SENP2, among the six known SUMO proteases, as the major SUMO protease for NEMO [39]. This study also provided a new concept that SUMO and NF-*κ*B ties are not simply one way but can be bidirectional, SUMO regulating NF-*κ*B signaling on one hand and NF-*κ*B modulating SUMOylation via induction of SUMO proteases on the other, thereby implicating new roles for the NF-*κ*B system.

**Figure 3:**
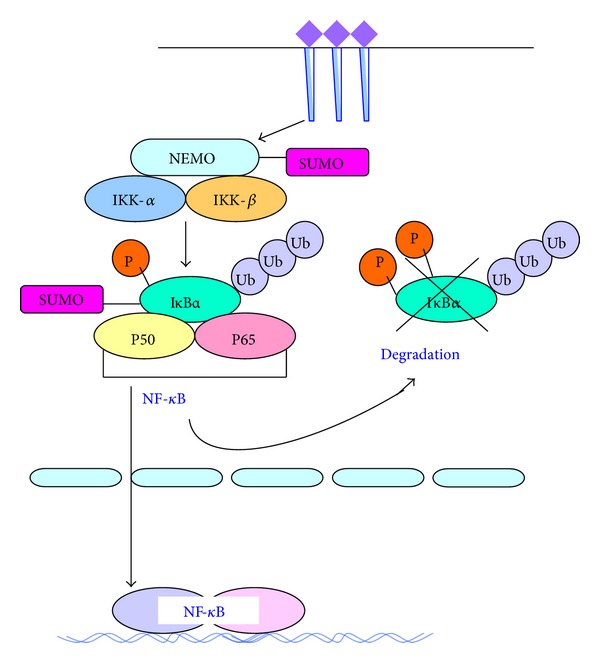
Regulation of NF-*κ*B signaling pathway by SUMO. Multiple signal transduction molecules of NF-*κ*B pathway, such as I*κ*B*α*, NEMO, RelA, and P100, can be modified by SUMO. After SUMO-1 conjugated to NEMO, the IKK would be activated, similarly, SUMOylation I*κ*B*α* conjugated to the SUMO-1, and this would lead to the degradation of I*κ*B*α* and activation of the NF-*κ*B pathway. And the SUMOylation of RelA and P100 was in accord with NEMO.

NF-*κ*B is regulated by SUMOylation, where the RelA subunit of NF-*κ*B is SUMOylated by PIAS3 [40]. PIAS3-mediated NF-*κ*B repression was compromised by either RelA mutant resistant to SUMOylation or PIAS3 mutant defective in SUMOylation. PIAS3-mediated SUMOylation of endogenous RelA was induced by NF-*κ*B activation thus forming a negative regulatory loop. The SUMOylation of endogenous RelA was enhanced in I*κ*B*α* null as compared with wild type fibroblasts. The RelA SUMOylation was induced by TNF*α* but not leptomycin B mediated RelA nuclear translocation. Furthermore, RelA mutants defective in DNA binding were not SUMOylated by PIAS3, suggesting that RelA DNA binding is a signal for PIAS3-mediated SUMOylation (Figure 3).

### 5.2. Ubiquitination, SUMO, and Transforming Growth Factor *β* (TGF-*β*)

Glomerular sclerosis and interstitial fibrosis are the major pathological changes of advanced diabetic nephropathy; transforming growth factor *β* (TGF-*β*) is a key factor in renal fibrosis of DN [41]. Studies showed that high glucose, angiotensin II (Ang II), and other profibrotic factor were involved in the activation of TGF-*β* pathway [42, 43]. TGF-*β* plays an important role in diabetic nephropathy fibrosis, TGF-*β* induced glomerular and tubular cell hypertrophy, extracellular matrix (ECM) accumulation, the promotion of glomerular sclerosis, and renal interstitial fibrosis.

The multifunctional proteins TGF-*β*s, activin, and bone morphogenetic proteins (BMPs) are the members of TGF-*β* family, which regulate a wide variety of cellular responses, for example, proliferation, differentiation, migration, and apoptosis. Smad protein is an important signaling molecule and mainly negative controlling protein in TGF-*β* downstream [44]. This signaling is tightly regulated by various posttranslational modifications including ubiquitination. Type I TGF-*β* receptor (TGF-*β*R I) is degraded by Smad7-dependent ubiquitination-proteasomal pathway, which is deubiquitinated by ubiquitin C-terminal hydrolase-L5 (UCHL5). UCHL5 is required for high glucose-induced TGF-*β*R I protein expression and deubiquitination in mesangial cells and for fibronectin expression and cell hypertrophy [42]. Several E3 ubiquitin ligases play a crucial role in the specific recognition and ubiquitin-dependent degradation of Smad [45].

Smad ubiquitination regulatory factor (smurf) is the specific ubiquitin ligase of Smad degradation, which disclosed Smad signal pathway terminating in the ubiquitin proteasome degradation. Smurf1 and Smurf2 are the two members of the Smurf; Smurf1 can directly bind to Smad1, degrade Smad1, Smad5, and Smad7; Smurf2 could combine with Smad7 and the complexes of TGF-*β* receptor and that would result in the degradation of TGF-*β* receptor complexes and Smad7; the inhibition of TGF-*β* signal by Smad7 weakened, thus TGF-*β* signal enhanced. In the tubules interstitial fibrosis mouse model after unilateral ureteral obstruction, Asano et al. found the expression of Smad7 reduced, but Smad7 mRNA expression did not decrease in the kidney, and increased ubiquitin degradation of Smurf1/2 led to low expression of Smad7 protein, which suggested the degradation of Smad7 by UPS is important for the fibrosis of renal tubule interstitial [46]. The same results have also been found in the studies of scleroderma skin fibrosis and liver fibrosis, so feedback signal of TGF-*β* Smad reduced with the degradation of Smad7 by UPS increased, which was an important mechanism of organ fibrosis.

Our recent studies found the ubiquitination of histone H2A and deubiquitination of histone H2B in glomerular mesangial cells could activate TGF-*β* signaling pathway, which is involved in the pathogenesis of diabetic nephropathy [47]. In glomerular mesangial cells induced by high glucose for 6~24 hours, the expression of Smurf2 and Smad2/3 increased and on the contrary the expression of Smad7 decreased, so TGF-*β* signaling pathway was activated, which would induce the secretion of extracellular matrix protein FN and lead to fibrosis of diabetic nephropathy [48–50]. We also detected that Smad7 protein expression decreased in DN rats, but Smurf2 and FN mRNA expression and TGF-*β* protein expression increased [51].

In recent years, the role of SUMOylation in regulation of TGF-*β* signaling becomes the focus of research; the present study supports that the Smad protein SUMOylation inhibits the transcriptional activity, but SUMOylation of TGF receptor increased the affinity with its ligand. Smad3 and Smad4 play crucial roles in transforming growth factor-beta- (TGF-*β*-) mediated signaling pathway, which produce a variety of cellular responses, including cell proliferation and differentiation. A previous study demonstrated that PIASy suppresses TGF-*β* signaling by interacting with and sumoylating Smad3. Sumoylation of Smad4 regulated its stability. The present study found coexpression of Smad3 with PIASy and SUMO1 stimulated the nuclear export of Smad3 [52]. Mutation of the Smad4 sumoylation sites or cotransfection with SuPr-1 greatly increases Smad4 transcriptional activity. Moreover, direct fusion of SUMO-1 to the sumoylation mutant Smad4 potently inhibits its transcriptional activity [53]. These results suggest that PIASy regulates TGF-beta/Smad3-mediated signaling by stimulating sumoylation and nuclear export of Smad3. Sumoylation also represses Smad4 transcriptional activity.

SUMO can conjugate to cell-surface receptors for growth factors to regulate their functions [54]. Studies show that the type I transforming growth factor-*β* (TGF-*β*) receptor, T*β*RI, is sumoylated in response to TGF-*β* and that its sumoylation requires the kinase activities of both T*β*RI and the type II TGF-*β* receptor, T*β*RII. Sumoylation of T*β*RI enhances receptor function by facilitating the recruitment and phosphorylation of Smad3, consequently regulating TGF-*β*-induced transcription and growth inhibition. T*β*RI sumoylation modulates the dissemination of transformed cells in a mouse model of T*β*RI-stimulated metastasis. T*β*RI sumoylation therefore controls responsiveness to TGF-*β*, with implications for tumour progression.

Recent progress has been made on the role of oncoproteins c-Ski and related SnoN in the control of cellular transformation. c-Ski/SnoN potently repress TGF-*β* antiproliferative signaling through physical interaction with signal transducers called Smads. SnoN is modified by small ubiquitin-like modifier-1 (SUMO-1) [55]. Sumoylation occurs primarily at lysine 50 (Lys-50). PIAS1 and PIASx serve as SUMO-protein isopeptide ligases (E3) for SnoN sumoylation. SnoN sumoylation does not alter its metabolic stability or its ability to repress TGF-beta signaling.

Arkadia is a RING domain E3 ubiquitin ligase that activates TGF-*β* pathway by inducing degradation of the inhibitor SnoN/Ski. Studies showed evidence that Arkadia can function as a SUMO-targeted ubiquitin ligase (STUBL) by ubiquitinating SUMO chains. While the SIMs of Arkadia are not essential for SnoN/Ski degradation in response to TGF-*β*, they are necessary for the interaction of Arkadia with polysumoylated PML in response to arsenic and its concomitant accumulation into PML nuclear bodies [56], suggesting Arkadia to be a novel STUBL that can trigger degradation of signal-induced polysumoylated proteins.

### 5.3. Ubiquitination, SUMO, and Nrf2-Oxidative Stress

Oxidative stress may play an important role in the pathogenesis of diabetic nephropathy (DN). Nrf2 as a transcription factor is a valuable therapeutic target for prevention of oxidative stress and damage for regulating the expression of a group of antioxidant genes [19, 20]. In the nucleus, Nrf2 increases gene expression of antioxidant enzymes such as superoxide dismutase (SOD), glutathione peroxidase (GPx), catalase (CAT), and NAD (P) H [57–59].

Recent studies have shown that the ubiquitin-proteasome pathway (UPP) and oxidative stress have interaction. Inhibitor of UPS MG132 upregulated antioxidant genes and had a preventive effect on DN development and progression in rats [60]. In diabetic patients, oxidative stress can also target kidney by impairing UPS activity, which, caused by hydrogen peroxide (H_2_O_2_), inhibited proteasome activity and increased the levels of ubiquitin proteins [61]. Oxidative stress is closely touched with the regulation of proteasome's proteolytic activity. Kelch-like ECH-associated protein 1 (Keap1) is known as an actin cytoskeleton-associated protein; it always binds very tightly to Nrf2 in the cytoplasm [62] and serves as a substrate adaptor for Cullin-3 to form the E3 ubiquitin-ligase complex, which ultimately leads to ubiquitin degradation of Nrf2 [63].

PA28 protein is 11S regulatory subunits of the mammalian ubiquitin proteasome. Hyperglycemia regulates UPS activity in vascular units of the glomerulus. Researches detected that increased level of PA28 proteins in the glomerulus also synchronized with oxidative stress in DN, which might play a protective role against oxidative damage [64, 65]. Furthermore, the initial activation of PA28 proteins may protect glomerulus from oxidative stress induced by hyperglycemia, but the chronic activation of PA28 proteins would exacerbate the pathogenesis of DN [26]. Luo et al. found that the activity of 26S proteasome in the kidney of DN rats is 3.68 times greater than that of normal control rats at 12 weeks. At the same time, the activity of superoxide dismutase (SOD) and glutathione peroxidase (GPX) decreased significantly, which would weaken the ability of scavenging oxyradical and thereby the oxidative stress of kidney increased [66].

### 5.4. Ubiquitination, SUMO, and MAPK

The MAPK pathways are one of the important routes by which extracellular signals are transduced into intracellular responses. Through protein phosphorylation mechanisms, they can play a pivotal role in regulating other posttranslational modifications such as protein acetylation and ubiquitination. Previously, a study found that Ste7, a prototype MAPKK in yeast, is ubiquitinated upon pheromone stimulation; ubiquitin ligase SCF (Cdc4) and the ubiquitin protease Ubp3 have opposing effects on Ste7 ubiquitination, even SCF (Cdc4) is necessary for proper activation of the pheromone MAPK Fus3, and Ubp3 is needed to limit activation of the invasive growth MAPK Kss1 [67]. MKP-1 is a specific negative regulator of MAPK signaling pathway, which has promised to inactivate MAPK by regulating the dephosphorylation of MAPK. Studies have found that MKP-1 was regulated by ubiquitin degradation of UPS [68].

Our experiment detected that ubiquitin degradation of MKP-1 increased in DN rats, and MG132 as an ubiquitin proteasome inhibitor can inhibit the degradation of MKP-1 and block the activation of the MAPK pathway [69].

In addition, protein sumoylation has emerged as an important pathway which also functions through posttranslational modification. The SUMO pathway modulates a diverse range of cellular processes including signal transduction, chromosome integrity, and transcription. Interestingly, recent studies have provided links between the SUMO and MAPK signaling pathways which converge to modulate transcription factor activity [70]. This was first demonstrated by the observation that the activation of the ERK pathway caused desumoylation of the transcription factor, Elk-1 [71]. Furthermore, a growing number of links are now being made between the MAPK pathway and protein sumoylation. SUMO covalently attaches to certain residues of specific target transcription factors and could inhibit its activity, suggesting the involvement of ERK5 SUMOylation on its transcriptional activity. Point-mutation analyses showed that ERK5 is covalently modified by SUMO at 2 conserved sites, Lys6 and Lys22, and small interfering RNA PIAS1 reversed H(2)O(2) and AGE-mediated reduction of shear stress-mediated ERK5/myocyte enhancer factor 2 transcriptional activity [72]. These data defined SUMOylation-dependent ERK5 transcriptional repression independent of kinase activity and suggested this process as among the molecular mechanisms of diabetes-mediated endothelial dysfunction. Given the nature of protein sumoylation in diverse biological functions, it is not surprising that the effect of MAPK pathways on sumoylation varies between different proteins.

Tissue transglutaminase (TG2), a multifunctional enzyme critical to several diseases, is constitutively upregulated in driving chronic inflammation. Alessandro Luciani [73] demonstrates that the generation of an oxidative stress induced by CFTR-defective function leads to protein inhibitor of activated STAT (PIAS) y-mediated TG2 SUMOylation and inhibits TG2 ubiquitination and proteasome degradation, leading to sustained TG2 activation. This PPAR and I*κ*B*α* SUMOylation leads to NF-KB activation and to an uncontrolled inflammatory response. TG2 may function as a link between oxidative stress and inflammation by driving the decision as to whether a protein should undergo SUMO-mediated regulation or degradation. Targeting TG2-SUMO interactions might represent a new option to control disease evolution in CF patients as well as in other chronic inflammatory diseases, such as DN.

## 6. Ubiquitination, SUMO, and the Treatment of DN

Nowadays, the treatment of DN is limited, unsatisfactory, and expensive. UPS is involved in the progress of DN by regulating several signaling pathways, such as NF-*κ*B, TGF-*β*, Nrf2-oxidative stress, and MAPK. MG132, a proteasome inhibitor, was constructed by Lee et al. in 1994 and has been widely used in studying the treatment of DN [74, 75]. Research has shown that MG132 has therapeutic effects on DN [47, 60, 76], but the mechanism by which it acts is unclear.

**Figure 4:**
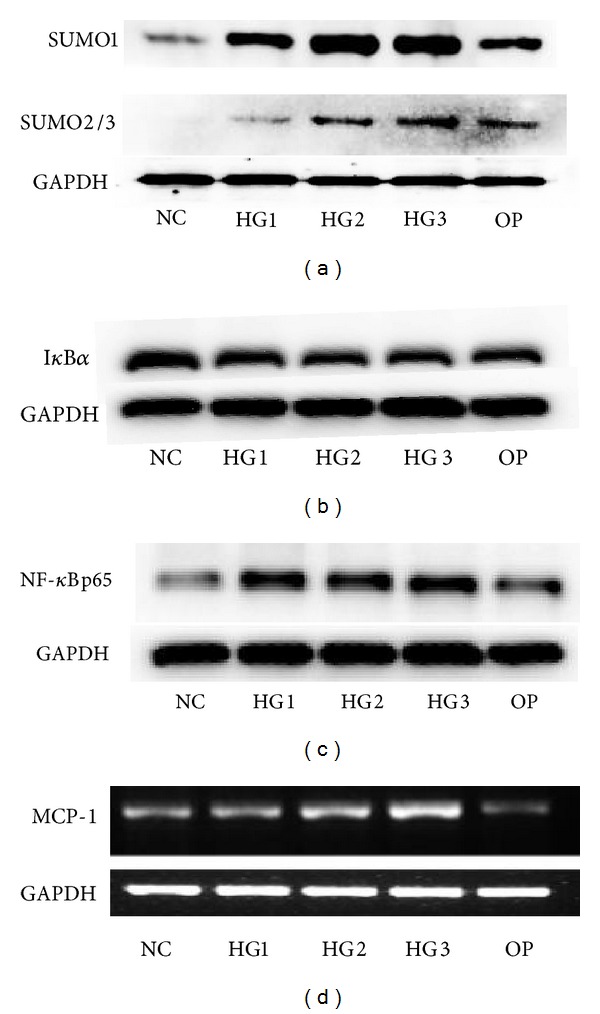
The result of our experiments. (a) SUMO1 and SUMO2/3 protein expression after various glucose concentrations' challenge determined by Western blot. (b) I*κ*B*α* protein expression after various glucose concentrations' challenge determined by Western blot. (c) NF-*κ*Bp65 protein expression after various glucose concentrations' challenge determined by Western blot. (d) MCP-1 mRNA expression after various glucose concentrations' challenge determined by RT-PCR. The expression of SUMO1 and SUMO2/3 enhanced in rat glomerular mesangial cells induced by high glucose, while I*κ*B*α* sumoylation decreased significantly, but NF-*κ*Bp65 and MCP-1 were increased under high glucose conditions.

Reports exposed that MG132 prevents NF-kB activation by inhibiting ubiquitin proteasome specifically in experimental diabetes and DN, and the typical features of DN, such as inflammation, proteinuria, basement membrane thickening, and glomerular mesangial expansion were improved obviously after the treatment of MG132 in diabetic mice [59, 77]. In our experiments, we detected that the expression of SUMO1 and SUMO2/3 enhanced in rat glomerular mesangial cells induced by high glucose, while I*κ*B*α* sumoylation decreased significantly, but NF-*κ*Bp65 and MCP-1 were increased under high glucose conditions, and all the changes can be reversed by adding MG132 (Figure 4) [35]. So, the inhibition of UPS is enough to alleviate the adverse effects of NF-kB activation on the development of DN.

TGF-*β* is a therapeutic target for renal fibrosis. Scientists have long sought ways to antagonize TGF-*β* to treat diabetic nephropathy. UPS can activate TGF-*β* signaling pathway by degrading negative protein Smad7. We found that MG132 as a ubiquitin proteasome could block ubiquitin degradation of Smad7 and inhibit activation of the TGF-*β* signaling pathway in DN rats [51].

Accumulating investigation has demonstrated that proteasome inhibitor MG132 could reduce degradation of ubiquitin conjugated Nrf2 by inhibiting activity of the subunits of the core particle of 26S proteasome and activating the Nrf2-ARE signaling pathway [76, 78–80]. Luo et al. provided experimental evidence indicating that Nrf2-ARE signaling pathway activation can be used therapeutically to alleviate renal damage induced by type 1 diabetes [60, 76].

Antifibrotic effect of MG132 at low doses has been observed in rat renal fibroblasts and mesangial cells [81, 82]. Luo et al. found MG132 can significantly decrease the 26S proteasome activity, increase SOD and GSH-PX activity in DN rats, and enhance the antioxidative ability to inhibit the oxidative damage of kidney in DN rats [66]. Through systemically analyzing dose-dependent effects of MG132 using human umbilical cord vein cells, Meiners et al. found that nontoxic MG132 might offer a new therapeutic approach for the treatment of oxidative stress-associated renal diseases, whereas high doses of MG132 (200 nM) induced apoptosis in endothelial cells [83]. In OVE26 diabetic mice, researchers also found that MG132 upregulated Nrf2 function via inhibiting the increased proteasomal activity, which provided therapeutic effects on the kidney against oxidative damage, fibrosis, and eventually dysfunction [60]. In addition, MG132 selectively upregulates PAI-1 expression and activates MAPK pathways as well as PI3 K/Akt pathways. Inhibitors of these signaling pathways reduced MG132-mediated upregulation of PAI-1 in varying degrees [84]. Therefore, MG132 has great potential as a therapeutic agent for DN.

In summary, ubiquitination and SUMO could activate NF-*κ*B, TGF-*β*, and MAPK and inhibit Nrf2-oxidative stress by degrading the related signal proteins to be involved in the progression of DN. Low dose of MG132 could reverse these changes and improve renal injury, and maybe it is a potent target for amelioration of DN patient. However, study of the treatment in DN still requires more research. Maybe, using a proteomic approach could study more substrates of ubiquitination and SUMO, which would reveal the new pathogenesis of DN. This may find new therapeutic target for DN and identified DN-related biomarkers earlier.
